# Effect of mechanical combined with electromagnetic stirring on the dispersity of carbon fibers in the aluminum matrix

**DOI:** 10.1038/s41598-020-64983-5

**Published:** 2020-05-15

**Authors:** Guanglong Li, Yingdong Qu, Yaohua Yang, Ruirun Chen, Qiwen Zhou, Rongde Li

**Affiliations:** 1 School of Materials Science and Engineering, Shenyang University of Technology, 110870 Shenyang, China; 2 School of Materials Science and Engineering, Harbin Institute of Technology, 150001 Harbin, China

**Keywords:** Composites, Design, synthesis and processing

## Abstract

In order to improve the uneven distribution of carbon fibers (CFs) in the matrix by traditional single mechanical stirring, mechanical combined with electromagnetic (M-E) stirring was used to prepare short carbon fibers reinforced aluminum matrix (Csf/Al) composites. The 3-D flow field of aluminum melt under mechanical/M-E stirring were calculated and compared. The calculation results show that the complexity of flow field under M-E stirring could be significantly enhanced relative to a single mechanical stirring, especially there was a strong melt flow near the crucible wall due to the skin effect. It was found that except the inertial force under mechanical stirring and the melt collision with the crucible walls, CFs were also subjected to the electromagnetic force and the oscillating flow between the eddy currents, which would promote the dispersity of the short CFs in the composites. The experimental results are consistent with the calculation results. The experimental results show that the distribution of CFs at each position in the composite samples prepared under M-E stirring was stable. The uniform distribution of CFs in the composites would play an important role in improving the overall performance of the Csf/Al composites.

## Introduction

Carbon fibers reinforced aluminum matrix composites are regarded as promising structural and functional materials due to their high specific strength, good high temperature resistance and thermal conductivity^1^. Among them, the short carbon fibers reinforced aluminum matrix (Csf/Al) composites have wider application space because of its isotropic performance advantage^2^. However, short carbon fibers (CFs) have a certain aspect ratio and are different from particle reinforcements, so the dispersion of the CFs in the matrix limits the further improvement in the performance of composites and its application^3^. Although there are still many problems to be solved in the preparation of Csf/Al composites to achieve performance improvements^4–6^. The main purpose of this study is to improve the dispersion of CFs in the matrix and contribute to the development of composites through this basic research.

At present, although most of the preparation methods such as powder metallurgy^7^ or semi-solid methods^8^ could be used to prepare Csf/Al composites, fibers damage is easily caused by the weak shear resistance of the CFs. The mechanical stirring casting has the advantages of less fibers damage due to the CFs dispersion in the liquid state, while it has higher production efficiency and low cost compared to other methods. Naji *et al*.^9^ successfully prepared the Csf/Al composites by mechanical stirring, and the results show that the volume fraction and aspect ratio of CFs have a serious impact on the performance of the composites. The effects of carbon nanofibers dispersion on mechanical properties of composites were studied by Lim *et al*.^10^. The results show that the yield strength, ultimate tensile strength, elastic modulus, and micro-Vickers hardness of the composites would increase significantly with the addition of fibers, but the local agglomeration of CFs limited the further improvement in performance. Singh *et al*.^11^ also manufactured the carbon fiber reinforced aluminum matrix composites by a stirring process, but the distribution of fibers was inhomogeneous due to the agglomeration of the CFs. Li *et al*.^12^ used square crucible to prepare Csf/Al composites, which promoted the dispersion of CFs by increasing the turbulence intensity in the melt during the stirring process. At the same time, the effect of mechanical stirring speed on the dispersibility of CFs was also analyzed. With the increase of the stirring speed, higher turbulent kinetic energy in the melt will be beneficial to the dispersion of the short CFs. When the stirring speed reaches 1000 rpm, the dispersion effect of the short CFs in the matrix can achieve the best, but a small amount of fiber agglomeration still exist in the corners of the matrix. It can be known from the previous investigation that even though the Csf/Al composites can be prepared by mechanical stirring, the local agglomeration of CFs limits the performance of the composites. Therefore, in order to prepare composites with excellent performance, it is necessary to improve the traditional liquid mechanical stirring.

Electromagnetic stirring has been widely applied in the casting of molten metals with its high efficient and environment protection^13,14^. Li *et al*.^15^ used electromagnetic stirring technology to make the AZCa912 alloys effectively refined. Wei *et al*.^16^ studied the effect of permanent magnetic stirring on solidification of Al-4Cu and 2024 Al, and the results indicated that the magnetic stirring can obviously refined the grain due to it can lead to heat and mass transfer in the melt. Hachni *et al*.^17^ found that the forced convection caused by a traveling magnetic field could effectively reduce macrosegregations and promote equiaxed structures. Yang *et al*.^18^ reported that the non-uniform current density along radial direction due to skin effect would promote the melt have a strong axial flow. Therefore, in order to improve the dispersibility of CFs under mechanical stirring, the introduction of electromagnetic stirring is an ideal method.

In order to improve the distribution of CFs in Csf/Al composites by mechanical combined with electromagnetic (M-E) stirring process, it is necessary to have a better understanding of the details of melt flow. In this work, the 3-D mathematical model was established for calculating and comparing the fluid field under various processing parameters during mechanical and M-E stirring. The characteristics of the fluid flow in the square crucible were analyzed in detail. The Csf/Al composites were prepared under mechanical stirring and M-E stirring, respectively. The dispersion of the short CFs in the matrix and their tensile strength were examined.

## Results and Discussion

### Distribution of flow field in square cricible under mechanical stirring

**Figure 1:**
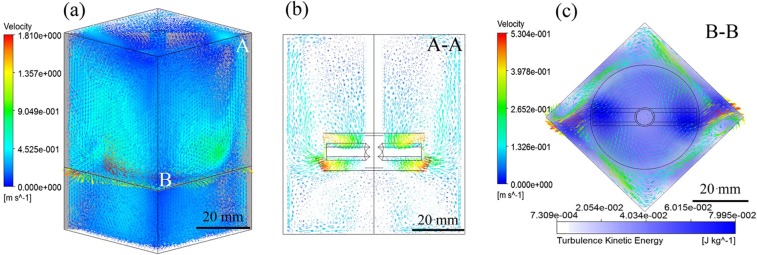
Calculated time-averaged velocity vectors in the melt under mechanical stirring (**a)** 3-D flow field in melt, (**b**) flow field in section A, (**c**) section B.

Liquid mechanical stirring method is an effective way to achieve dispersion^19^. Previous studies have shown that the dispersibility of CFs in square crucible under mechanical stirring with 1000 rpm is improved obviously compared with the sample prepared in circular crucible. The melt would constantly collide with the crucible walls inside the non-axisymmetric crucible under the inertial force of mechanical stirring, which would produce a complex three-dimensional flow field distribution and high turbulent kinetic energy in the crucible, as shown in Fig. 1. It is these violent collisions and convection between the vortices that promote the dispersion of CFs. But the non-axisymmetric structure is two-sided, it can be seen from Fig. 1(c) that small vortices appear in the crucible, the core of vortices located at the corner of the square crucible due to the right angle structure hinders the melt flow. This will cause the CFs to accumulate with the flow at the corners, and the flow velocity at the core approaches zero, which will cause the CFs to agglomerate at the corners. As well as, it can be seen from the distribution of the turbulent kinetic energy that the local turbulent kinetic energy value is high in the melt due to the collision and convection of the melt, but the value at the vortex in the corner is very low. The low turbulence intensity during mechanical stirring also can not effectively promote the uniform distribution of CFs. Therefore, in order to achieve the uniform distribution of CFs in the matrix, it is necessary to improve the distribution of flow field inside the melt.

### Electromagnetically driven flow and mass transfer behaviors

**Figure 2:**
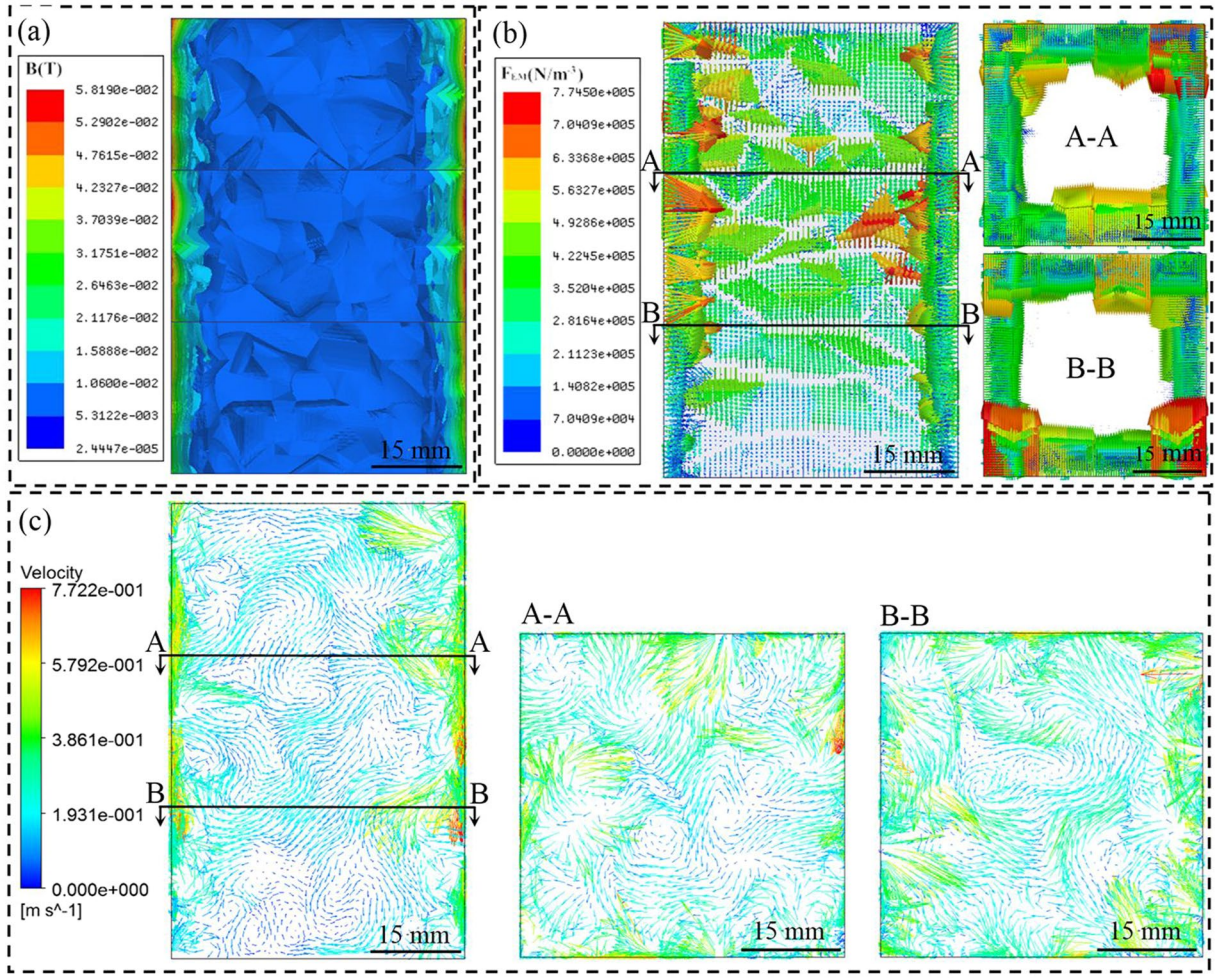
Magnetic flux density, electromagnetic force and flow field distribution in the crucible (**a**) magnetic flux density contours at the vertical section, (**b**) electromagnetic force vectors at the vertical section and cross section, (**c**) flow field distribution at the vertical section and cross section.

**Figure 3:**
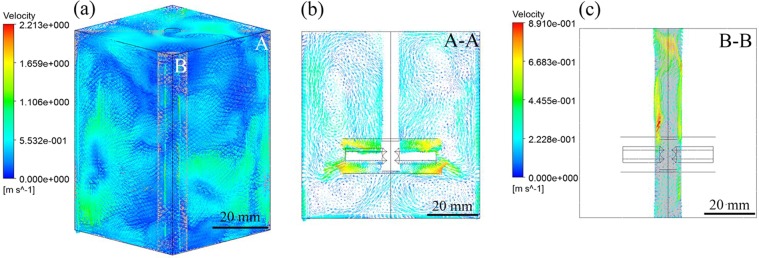
Calculated time-averaged velocity vectors in the melt under M-E stirring (**a**) 3-D flow field in melt, (**b**) flow field in section A, (**c**) section B.

According to the theory of electromagnetic stirring, the electromagnetic force would drive the melt to produce circulating flow in the crucible. The distribution of magnetic flux density (B) and electromagnetic force (F_EM_) in the crucible are shown in Fig. 2. In order to keep the melt at 1073 K during the experiment, the current through the induction coil is 900 A, so the current value in the simulation process is 900 A. It can be seen from Fig. 2(a) that the magnetic flux density is stronger at the surface and decreases towards the interior region due to the skin effect, which is consistent with Chen *et al*.^20^. At the same time, the magnetic flux density is uneven in the axial direction due to it is in the different position of the coil. The distribution of electromagnetic force is the same as magnetic flux density, which will result in a pressure gradient in the axial direction, as shown in Fig. 3(b). At the cross section, the strongest electromagnetic force is located at the surface and gradually decrease to the interior region due to the skin effect. The pressure gradient can be produced whether transverse and axial by electromagnetic stirring, which is beneficial to melt flow. In particular, there is a strong electromagnetic force at the corners in the square crucible, which has a positive effect on improving the problem of weak flow under the mechanical stirring.

Under the same conditions, the 3D mean fluid flow in the crucible is shown in Fig. 2(c). The electromagnetic force distributed along the crucible wall will induce a meridional flow in the melt^21^. It can be found that the flow of melt to the center of the crucible is driven by the maximum electromagnetic force at the crucible wall by comparing Fig. 2(b). The maximum velocity in this case is 0.77 m s^−1^ closing to the crucible walls due to skin effect. The flow will then recirculate back through the core of the melt, thus presenting an axisymmetric flow pattern inside the crucible. Meanwhile, several eddy currents can be seen in the distribution of the flow field. As reported by Umbrashko *et al*.^22^, the velocity oscillations would promote convective heat and solute transfer when two or more eddy currents are included in the flow structure. The local high velocity flow is also conducive to the transport behavior of CFs between eddies. At the cross section, there are many eddy currents in the melt. At section A-A, the strongest flow appears near the crucible walls and flows to the center due to the electromagnetic force gradient along the radial. The right angle region of the square crucible which is easy to hinder flow also has a strong melt flow due to there is a strong electromagnetic force here as discussed above. The flow pattern at section B–B is just like that at section A–A, which indicates that the flow pattern is holistic. Therefore, the addition of electromagnetic stirring during mechanical stirring is an ideal solution to further promote axial flow of the melt and uniform distribution of the CFs.

### Influence of M-E stirring on flow field distribution

The distribution of flow field in the crucible under M-E stirring at 1000 rpm and 900 A current is shown in Fig. 3. It can be seen that the flow field distribution is quite different from that of mechanical stirring in Fig. 1. The flow field distribution under M-E stirring will not only retain the characteristics of the flow field under mechanical stirring, but also increase the flow field distribution under electromagnetic stirring. Due to the interaction between mechanical stirring and electromagnetic stirring, the flow velocity of melt is significantly increased compared with that under single mechanical stirring, and the complexity of the three-dimensional flow is also improved as can be seen from Fig. 3.

At section A-A, except the stable circulation flow in the melt, the number of eddy currents in the flow field under M-E stirring can be seen. The axial flow is obviously higher than that in mechanical stirring on the section A-A in Fig. 1(b). In this case, the problem of insufficient axial flow under single mechanical stirring can be effectively solved in theory. The introduction of more eddy currents is beneficial to the distribution of solute^18^. At section B-B in Fig. 3(c), the melt has a strong axial flow near the crucible wall at the corners due to skin effect, which is conducive to the migration of CFs at the edge of the melt and in the dead zone of the right angle region. Therefore, it can be found that except the inertial force under mechanical stirring and the melt collision with the crucible walls, there are also the electromagnetic force and the oscillating flow between the eddy currents inside the melt under the M-E stirring. The electromagnetic force and the oscillating flow between the eddy currents could further promote CFs distribution inside the melt. Therefore, from the viewpoint of flow field distribution, M-E stirring can promote the uniform distribution of CFs more effectively.

**Figure 4:**
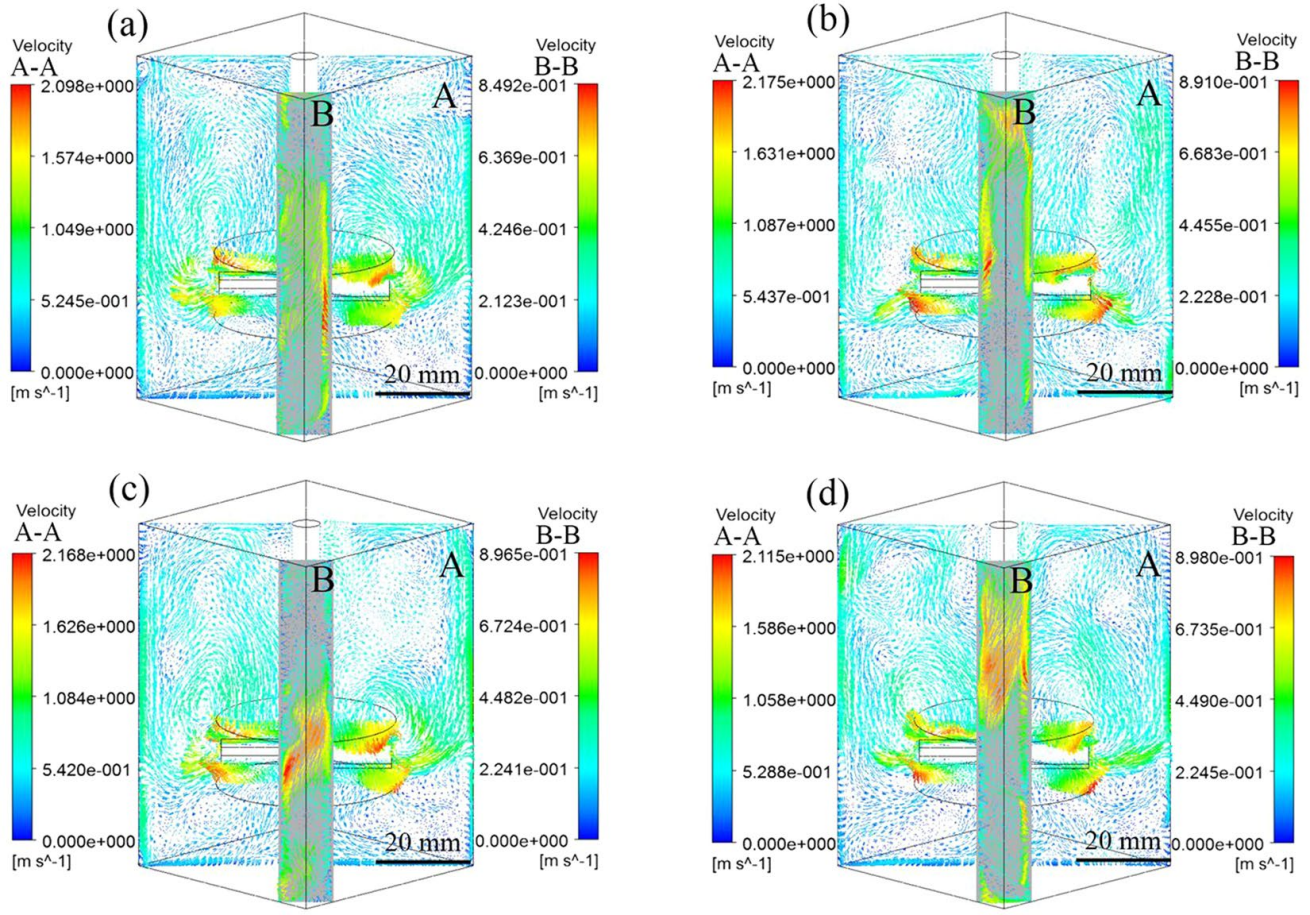
Flow fields under M-E stirring with different current (**a**) 850 A, (**b**) 900 A, (**c**) 950 A, (**d**) 1000 A.

During the M-E stirring process, the stirring speed and current intensity will seriously affect the flow state of melt in the crucible. Li *et al*.^12^ have studied the effect of the stirring speed on the fibers dispersion in square crucible, and found that the stirring speed of 1000 rpm was the most reasonable. Therefore, the influence of the change of electromagnetic field on the melt flow in the square crucible needs further study. The distribution of flow field in the crucible under M-E stirring at different current conditions are shown in Fig. 4. It can be seen that the distribution of the flow field in the crucible did not change obviously with the increase of current. The maximum velocity of melt flow also hardly changed at section A-A, as shown in Fig. 4. The previous research found that the melt flow rate under single mechanical stirring was much higher than that of single electromagnetic stirring, so mechanical stirring provided a larger driving force and had a greater impact on the melt flow. The introduction of electromagnetic field is to improve the flow of the melt in the right angle region of the square crucible. The axial flow is obviously on the section B-B in Fig. 4, but the flow velocity has only slightly increased with the current increases. Therefore, the increase of current has no significant effect on the melt flow in the square crucible. Akbarzadeh *et al*.^23^ found that high temperature could easily cause the reaction between CFs and melt to affect the properties of the composites. The experiment found that the current value of 900 A could ensure that the melt in a reasonable temperature range. Therefore, the theoretical calculation results show that the mechanical stirring speed of 1000 rpm and the current value of 900 A are reasonable parameters for M-E stirring.

### Carbon fibers distribution in the composites prepared under mechanical stirring and M-E stirring

**Figure 5:**
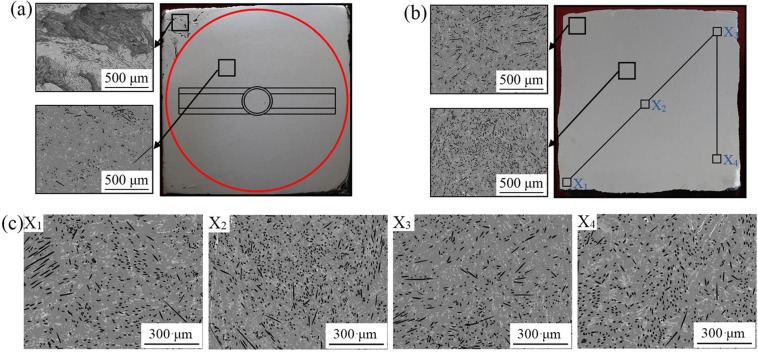
Macroscopic morphology and microstructure of the composites (**a**) prepared with mechanical stirring, (**b**) prepared with M-E stirring, (**c**) distribution of short CFs in the composites prepared by M-E stirring.

Figure 5 shows the macroscopic photographs and microstructure of the cross section of the samples prepared by mechanical stirring and M-E stirring, respectively (composite sample with a size of 48.7 mm*48.7 mm*75 mm). It can be seen from Fig. 5(a) that there is no defect in the central region (inside the red circle) of the sample prepared by mechanical stirring. In this area, the violent collisions and convection of the melt caused by the action of the stirring paddle and the vertical crucible wall will obviously promote the dispersion of the fibers, which is consistent with the calculation results of Fig. 1(c). However, there are obvious defects at the corner of the sample. It can be found from the magnification that these defects are caused by the un-infiltrated fiber clusters. Due to the existence of stirred dead zone in the non-axisymmetric right angle structure, the dispersion effect is obviously reduced at the corners. These will have a certain impact on fibers dispersion, which limits the overall performance of the composites. Therefore, in order to achieve the uniform distribution of CFs in the matrix, it is necessary to improve the fluidity of the melt and thus the dispersibility of the fibers.

As shown in the macroscopic morphology of Fig. 5(b), no obvious defects can be seen on the sample prepared by square crucible under M-E stirring. Especially in the corners of the sample, the defects are significantly improved compared to the sample prepared under mechanical stirring. As shown in the magnification diagram of Fig. 5(b), it can be seen that the short CFs can be uniformly dispersed both in the central region and at the coners of the sample, and the phenomenon of local agglomeration is greatly improved compared with that of single mechanical stirring. In order to further analyze the dispersion effect of M-E stirring on CFs, sampling and observation at random four positions in the sample prepared by M-E stirring, it can be seen that the uniform distribution of the short CFs in the matrix are shown in Fig. 5(c). Because of the inertia force under mechanical stirring, the short CFs along with the melt will continuously impact the end walls in the square crucible, which results in the initial dispersion of the short CFs. However, as the melt circulates in the radial direction, some of the CFs will accumulate in the right angle region where the stirring is weak. At this point, electromagnetic stirring will play an important role in promoting fiber dispersion. Electromagnetic force drives the melt to produce several axial annular flows. Especially near the crucible wall, the high velocity flow of melt will cause the fibers in the right angle region to flow along with the melt to the interior region. As Scepanskis *et al*.^24^ found that the strong oscillations of flow between the upper and the lower eddies promoted the solute transfer, such oscillating exchange of the particles between the eddy zones would lead to the homogenization of the particles in axial. In our case, at least three vortexes of the mean flow exist in the half X-Z plane of the melt as shown in Fig. 3, and the high frequency of alternating current would produce higher turbulent fluctuations^25^, hence the electromagnetic stirring can further promote the uniform distribution of CFs. Therefore, the coupling of the inertial force, the violent collision with the crucible wall, the electromagnetic force, and the oscillating flow between the eddy currents under M-E stirring is beneficial to the uniform dispersion of the fibers.

Previous study^26^ have confirmed that the fibers present in the matrix with un-infiltration or non-uniform distribution would significantly affected the comprehensive properties of composites. Therefore, the M-E stirring can obviously promote the uniformity of performance and significantly improve the performance and stability of castings.

## Conclusion

In summary, the effects of M-E stirring on short CFs distribution in the matrix of the Csf/Al composites were studied by numerical calculation and experiments. Compared with the distribution of flow field in the melt under mechanical stirring, M-E stirring enhances the complexity of axial flow field and promotes the transfer of solutes, especially there is a strong melt longitudinal flow near the crucible wall due to the skin effect. The theoretical calculationr esults show that the mechanical stirring speed of 1000 rpm and the current value of 900 A are reasonable parameters for M-E stirring. In the process of uniform dispersion of carbon fibers, the CFs are not only subjected to the inertial force of mechanical stirring and the collision with the crucible walls, but also to the electromagnetic force and the oscillating flow between the eddy currents under M-E stirring. In this case, CFs can be uniformly distributed in the casting, which can significantly improve the stability and the overall performance of the Csf/Al composites.

### Experimental procedure and mathematical models

**Figure 6:**
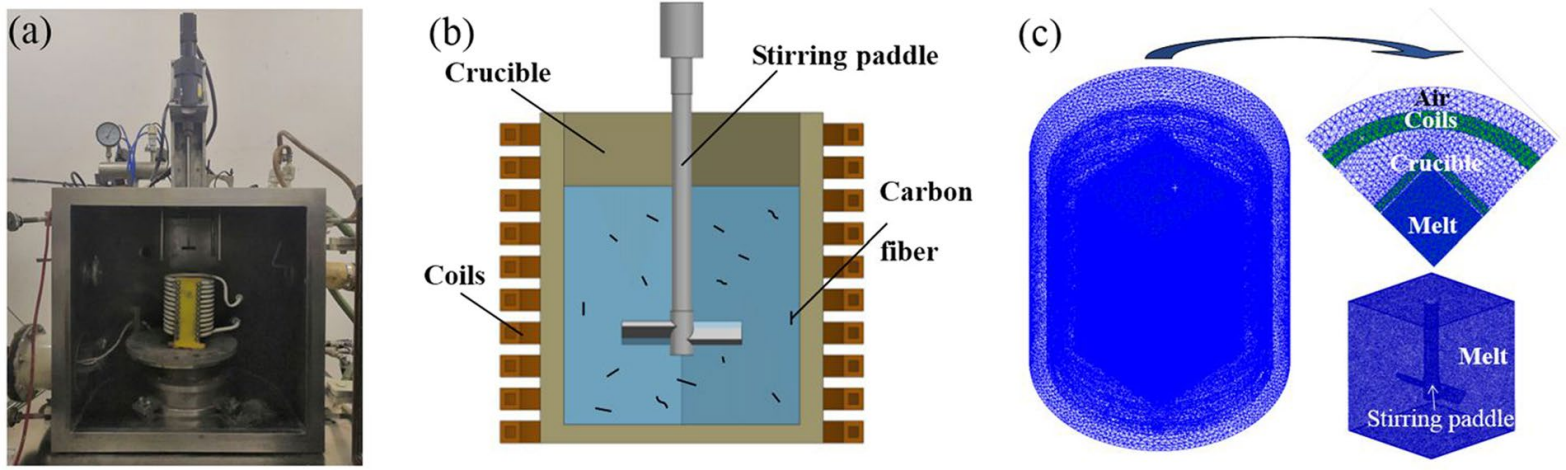
Experimental setup and simplified model (**a**) experimental setup (**b**) M-E stirring model and (**c**) finite element mesh.

The experimental setup is shown in Fig. 6(a). In order to achieve better mixing effect, this experiment used square crucible for alloy melting. The matrix material was Al-Si alloy and the reinforcement material was Polyacrylonitrile-based CFs (T-300) with an aspect ratio of 800. The melting, mixing and dispersing process were carried out in a vacuum chamber. After the aluminum alloy was completely melted, the melt was stirred and the CFs were dispersed. When the Csf/Al composites were prepared by single mechanical stirring, the experimental temperature was selected at 1027 K and the rotational velocity of mechanical stirring was 1000 rpm. Previous studies have shown that CFs has the best dispersion effect in the melt when the stirring speed is 1000 rpm^12^. The CFs were gradually added to the melt through the feeding device during mechanical stirring. When the Csf/Al composites were prepared by M-E stirring, the current in the induction coil should be kept at 900 A (4 kHz) on the basis of mechanical stirring. Finally, the melt was cooled quickly to obtain the composite sample.

TM3030 scanning electron microscopy was used to investigate the distribution of the short CFs in the Csf/Al composites. The tensile testing was conducted on the electromechanical universal testing machine(MTS E45) under the loading rate of 0.5 mm/min at the atmospheric temperature.

The 3-D model of the experimental equipment and the mesh are shown in Fig. 6(b,c). The flow field of melt during mechanical stirring and M-E stirring processes were calculated by the program ANSYS (distributed by ANSYS HIT) using the two-way coupling method^18^. There were 722186 elements in the whole domain of the crucible for solving the flow field under mechanical stirring by Fluent and 830388 elements for solving the flow field under M-E stirring. The k-ɛ two-equation model was used to solve the turbulent flow. During the simulation calculation, the rotation velocity of stirring paddle is 1000 rpm and the material properties are shown in Table 1.

**Table 1:** Materials properties for simulation.

Parts	Coils	Aluminum melt	Surroundings (air)
Relative permeability	1	1	1
Resistivity (Ω m)	1.67×10^−8^	2.83×10^−8^	—
Permittivity	—	—	1.0059
Density (kg/m^3^)	—	2350	—
Dynamic viscosity (Pa s)	—	0.0012	—

The Navier-Stokes (N-S) equation used to accurately characterize the 3-D flow in the melt can be written as follows:

Continuity equation:

1 $$\nabla \cdot \rho \mathop{\to }\limits_{v}=0$$

Momentum equation:

2 $$\frac{\partial (\rho \mathop{\to }\limits_{v})}{\partial t}+(\rho \mathop{\to }\limits_{v}\cdot \nabla )=\upsilon {\nabla }^{2}\mathop{\to }\limits_{v}-\nabla p+{\mathop{\to }\limits_{F}}_{EM}$$

where *ρ* is the density, *v* is the dynamical viscosity and *p* is the pressure.
